# Leaf functional traits differentiation in relation to covering materials of urban tree pits

**DOI:** 10.1186/s12870-021-03316-8

**Published:** 2021-11-23

**Authors:** Jiyou Zhu, Yujuan Cao, Weijun He, Qing Xu, Chengyang Xu, Xinna Zhang

**Affiliations:** 1 Research Center for Urban Forestry, The Key Laboratory for Silviculture and Conservation of Ministry of Education, Key Laboratory for Silviculture and Forest Ecosystem of State Forestry and Grassland Administration, Beijing Forestry University, Beijing, 100083 China; 2 Research Institute of Tropical Forestry, Chinese Academy of Forestry, Guangzhou, 510520 Guangdong China

**Keywords:** Leaf functional traits, Adaptive strategies, Urban tree pits, Covering materials

## Abstract

**Background:**

Understanding the ecological strategies of urban trees to the urban environment is crucial to the selection and management of urban trees. However, it is still unclear whether urban tree pit cover will affect plant functional traits. Here, we study the response of urban trees to different tree pit covers, analyzed the effects of different cover types on soil properties and their trade-off strategies based on leaf functional traits.

**Results:**

We found that there were obvious differences in the physical properties of the soil in different tree pit covers. Under the different tree pit cover types, soil bulk density and soil porosity reached the maximum under cement cover and turf cover, respectively. We found that tree pit cover significantly affected the leaf properties of urban trees. Leaf thickness, chlorophyll content index and stomatal density were mainly affected by soil bulk density and non-capillary porosity in a positive direction, and were affected by soil total porosity and capillary porosity in a negative direction. Leaf dry matter content and stomata area were mainly negatively affected by soil bulk density and non-capillary porosity, and positively affected by soil total porosity and capillary porosity. Covering materials of tree pits promoted the functional adjustment of plants and form the best combination of functions.

**Conclusion:**

Under the influence of tree pit cover, plant have low specific leaf area, stomata density, high leaf thickness, chlorophyll content index, leaf dry matter content, leaf tissue density and stomata area, which belong to “quick investment-return” type in the leaf economics spectrum.

## Background

Street trees are an integral part of the urban green space system, and play a very important role in beautifying the city, improving the urban ecological environment, organizing urban traffic, and regulating the local climate of the city [1–3]. The microenvironment of the tree pit provides basic and significant conditions for the growth of urban street trees. It is an important space for street trees to absorb water and nutrients [4–7]. Urban tree pit plays an important role in protecting the water permeability and air permeability of the root system of street trees and the soil around the root system of street trees [8, 9]. Different tree pit treatment methods have different effects on the water and nutrient supply, urban beautification, urban management and pedestrian traffic [9, 10]. In the current global urban ecological construction boom, the selection and treatment of urban tree pit materials are of great significance to urban planning and urban tree management.

Studies have shown that hardened tree pits will cause soil compaction and poor ventilation, inhibit tree roots and soil microbial respiration [7, 11, 12]. Additionally, other studies have reported that plant coverage, ceramsite coverage and silica gel covers are beneficial to plant growth [13]. Therefore, from the perspective of plant growth requirements, different tree pit coverage types have different degrees of impact on plant growth. Reasonable treatment of street tree pits can not only protect the roots of trees from being trampled on, prevent the soil near the roots from being compacted, but also avoid raising dust, and protect the urban ecological environment [14]. However, the research on the effect of urban tree pit cover on plant functional traits is still limited. To fully comprehend the ecological effects of urban planning on plants, it is necessary to study their trade-off strategies under the urban tree pit covers. Plant functional trait is a vital bridge linking the environment and trees [15–17]. In previous studies, the research on the relationship between plant functional traits and the environment mainly involved temperature, precipitation, light, altitude, aspect, and geographical pattern [18–22]. However, we still lack a complete understanding of how leaf functional traits are shaped under the influence of the urban tree pit cover and how changes in leaf functional traits affect plant tolerance to urban tree pond cover and adjustment of trade-off strategies.

In this study, we selected the five most commonly used tree pit covering materials in Chinese cities, taking leaf functional traits as the starting point, and establishing the relationship between leaf functional traits and soil physical properties based on the response of leaf functional traits under different covering materials. It is expected to provide a scientific basis for the configuration, design and planning of urban tree pits.

## Results

### Leaf functional traits differentiation in tree pits treated with different covering materials

**Fig. 1:**
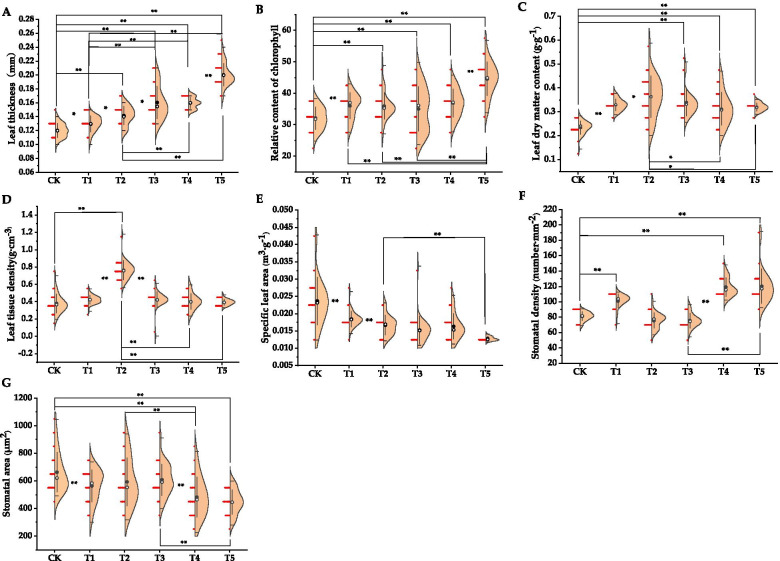
Changes in plant functional traits under different covering materials. **A** Leaf thickness, **B** Relative chlorophyll content, **C** Leaf dry matter content, **D** Leaf tissue density, **E** Specific leaf area, **F** Stomatal density, **G** Stomatal area. No cover (CK), turf (T1), permeable plastic blanket (T2), tree grate (T3), wood board (T4), cement (T5). “*” indicates that the significant level between treatments is at the *p* < 0.05 level, and “**” indicates that the significant level is reached between the treatments at *p* < 0.01. Same below.

As shown in Fig. 1, compared with CK, the leaf thickness of the 5 kinds of tree pits covered by the plant was significantly increased, and the leaf thickness values were cement > wooden board > tree grate > plastic blanket > CK. Similarly, chlorophyll content index of the plants with covered 5 tree pits all increased significantly, and the difference reached a very significant level. The chlorophyll content index values from high to low were cement > wood board > tree grate > plastic blanket > CK. Leaf dry matter content under the condition of covered tree pits was significantly greater than that of CK (plastic blanket > tree grate > turf > wood board > cement > CK). The leaf tissue density was plastic blanket> turf > tree grate > permeable wood board > cement > CK. The specific leaf area under different covering materials in descending order was CK > turf > plastic blanket > tree grate > wood board > cement. The stomata density in descending order was cement > permeable wood > turf > CK > plastic blanket >tree grate. The stomatal area under different covering materials was CK > turf > plastic blanket > tree grate > wood board > cement.

### Correlation between plant functional traits and urban soil physical properties

**Fig. 2:**
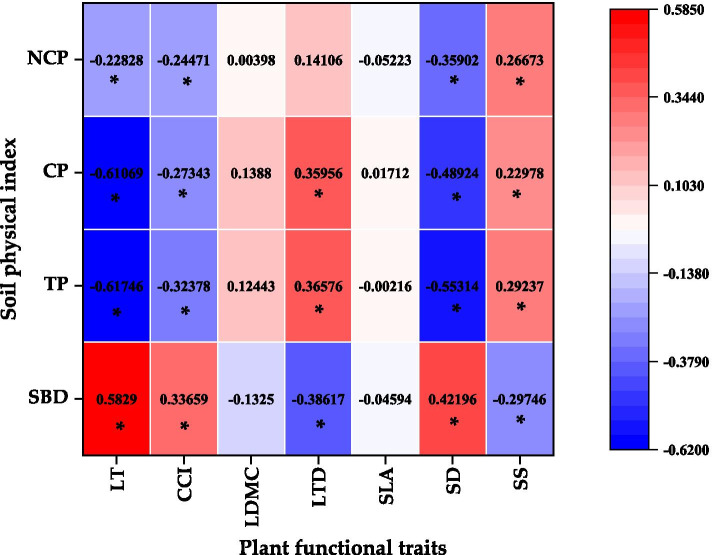
Heat map of correlation between plant functional traits and urban soil physical properties. SLA-Specific leaf area, LDMC-Leaf dry matter content, CCI-Chlorophyll content index, LTD-Leaf tissue density, LT-Leaf thickness, SD-Stomatal density, SA-Stomatal area

It can be seen from Fig. 2 that there was a significant positive correlation between soil bulk density and leaf thickness, chlorophyll content index and stomata density. There was a significant negative correlation between soil bulk density and leaf dry matter content and stomata area. There was a significant negative correlation between the total soil porosity and leaf thickness, chlorophyll content index and stomatal density. There was a significant positive correlation between the total soil porosity and leaf tissue density and stomatal area. There was a significant negative correlation between soil capillary porosity and leaf thickness, chlorophyll content index, and stomatal density. The capillary porosity has a significant positive correlation with leaf tissue density and stomatal area. Non-capillary porosity has a significant negative correlation with leaf thickness, chlorophyll content index and stomatal density. There was a significant positive correlation between non-capillary porosity and stomatal area.

### Correlation between leaf functional traits and its trade-off strategies

**Fig. 3:**
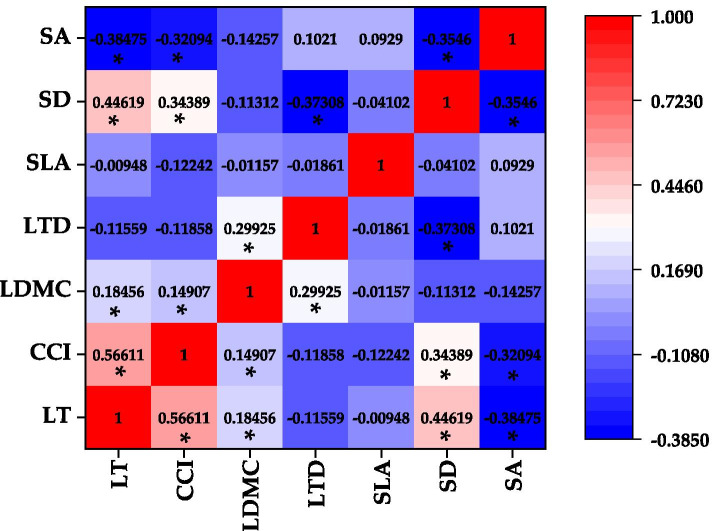
Correlation coefficients among leaf functional traits. SLA-Specific leaf area, LDMC-Leaf dry matter content, CCI-Chlorophyll content index, LTD-Leaf tissue density, LT-Leaf thickness, SD-Stomatal density, SA-Stomatal area

It can be seen from Fig. 3 that leaf thickness was positively correlated with chlorophyll content index, leaf dry matter content and stomatal density, and negatively correlated with stomatal area in the urban environment. Chlorophyll content index was significantly positively correlated with leaf dry matter content and stomata density, and was significantly negatively correlated with stomatal area. There was a significant positive correlation between leaf dry matter content and leaf tissue density. There was a significant negative correlation between leaf tissue density and stomatal density. There was a significant negative correlation between stomatal density and stomatal area.

**Fig. 4:**
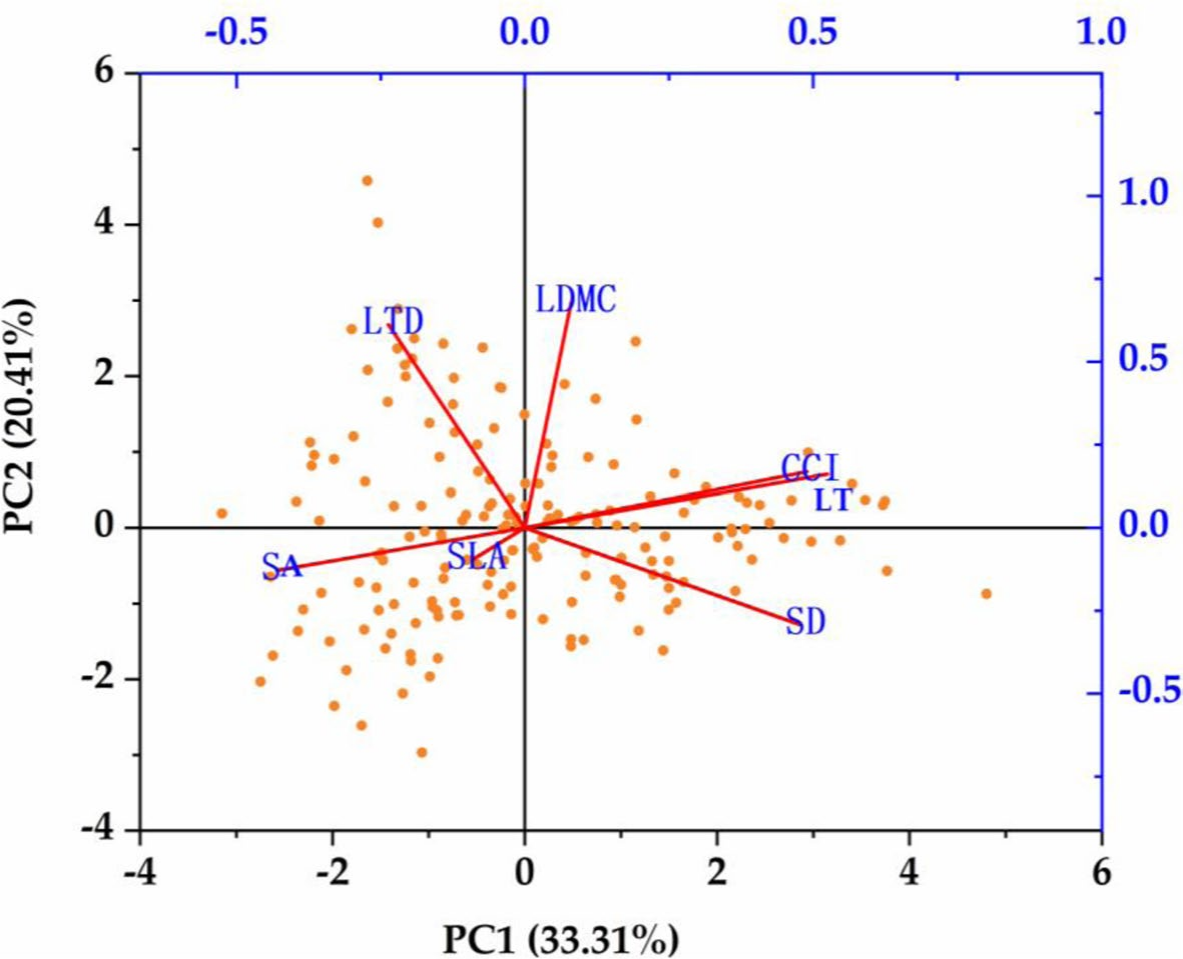
Principal component analysis biplot of leaf functional traits. SLA-Specific leaf area, LDMC-Leaf dry matter content, CCI-Chlorophyll content index, LTD-Leaf tissue density, LT-Leaf thickness, SD-Stomatal density, SA-Stomatal area

According to the principle that the eigenvalue is greater than 1, two principal components were extracted (the eigenvalues were 6.49 and 3.08). The contribution rates of these two principal components were 33.31 and 20.41% (Table 1). The cumulative contribution rate was 53.72%, indicating that these two principal components were the main factors for the change of leaf functional traits (Fig. 4). It can be seen that leaf thickness, chlorophyll content index, and stomata density were significantly positively correlated with the first principal component (Fig. 4). Leaf dry matter content, leaf tissue density has a significant positive correlation with the second principal component. Principal component analysis showed that the indicators (leaf thickness, chlorophyll content index and stomata density) that were significantly positively correlated with the first principal component could all be used as the main indicators of leaf functional traits.

**Table 1:** Factor matrix and principal component contribution rate of leaf functional traits

Traits	Ingredient score		Variance %	Accumulation %	Extract the sum of squared loads	
	PC1	PC2			Total	Accumulation %
LT	0.525	0.161	33.31	33.311	2.332	33.311
CCI	0.489	0.168	20.41	53.720	1.429	53.720
LDMC	0.080	0.678	14.34	68.056		
LTD	−0.237	0.612	10.26	78.313		
SLA	−0.091	− 0.096	8.68	86.988		
SD	0.479	−0.293	7.45	94.435		
SA	−0.430	−0.129	5.56	100.000

## Discussion

### Analysis of the difference in the physical properties of the tree pit soil treated with different covering materials

Covering tree pits with different covering materials can not only reduce rain erosion and surface runoff, but also lead to soil hardening and affect soil ventilation and permeability, thus having different effects on soil bulk density and porosity [23–25]. In this study, there were obvious differences in the influence of different covering materials on the physical properties of the tree pit soil. We found that the total soil porosity and capillary porosity of CK were both low. This may be closely related to pedestrian trampling or vehicle rolling, which will cause soil compaction, poor air permeability and water permeability [26]. On the contrary, if the cement was used to cover the soil completely, the soil bulk density will increase significantly and the soil porosity will decrease significantly. Cement covering not only causes the soil to be compact, but also severely hinders the penetration of external water and the exchange of external gases [27] Covering with tree grate can improve the pore structure of the soil, while reducing mechanical crushing and human trampling. Such covering materials can improve the permeability of the tree hole soil, significantly reduce the soil bulk density, increase the total soil porosity, and increase the capillary porosity and non-capillary porosity [28]. Although wood boards have better air permeability than cement, when the coverage area was too large, they will increase the possibility of soil compaction. The turf cover is easy to form a dense rhizome layer and a net-like turf, which not only can improve the permeability of the soil to a certain extent, but also is beneficial to the conservation of water and soil. Breathable plastic blanket has good water permeability. It can reduce surface evaporation, weaken ground runoff, and control surface temperature.

### Effects of different covering materials treatments on plant functional traits

The specific leaf area is closely related to the growth rate of plants. It can reflect the balance between the acquisition and utilization of carbon by plants [29, 30]. Plants with a lower leaf area put a large part of energy and nutrition to build a defense structure or increase the density of mesophyll cells to prevent excessive water loss [31, 32]. In this study, the specific leaf area of plants showed regular changes under different tree pit covers, and the specific leaf area was the smallest under the cover of cement material. The permeability of the soil under the cement cover was poor, causing the plants to be in a state of insufficient water supply for a long time. Therefore, plants in a cement-covered tree pit environment can reduce the impact of drought stress by reducing specific leaf area to prevent excessive water loss.

The increase in leaf thickness and density of mesophyll cells not only improves the utilization efficiency of light resources by leaves, but also enhances the protection effect of leaves on strong light [33–35]. In this study, there were significant differences in the effects of different covering materials on leaf thickness. Under the wood plank and cement cover, urban plants tend to choose the strategy of increasing leaf thickness, and put more dry matter to build defense organizations. By increasing the leaf thickness, plants increase the resistance to water transport and reduce the loss of water.

The chlorophyll content index is closely related to the photosynthetic capacity of plants [36, 37]. In this study, under the influence of covering materials such as wood planks and cement, soil air permeability and water permeability were reduced, which seriously hinders the absorption of water and nutrients by plant roots. Therefore, increasing the chlorophyll content index may be the balance of plant leaf and root level to resource absorption and utilization strategy. Plants further enhance their photosynthesis efficiency by increasing their chlorophyll content to produce more organic matter.

Studies have shown that the dry matter content of leaves is related to the nutrient retention capacity of plants, and can reflect the utilization status of plants to their habitat resources to a certain extent [38]. Leaf tissue density is closely related to the defense ability of plants [39, 40]. In materials with poor air and water permeability, such as cement and wood boards, the dry matter content and leaf tissue density of plants increase, which may be the self-protection of plants in an airtight and permeable environment. The purpose of increasing leaf dry matter content is to keep more nutrients to maintain normal plant growth. Increasing the density of leaf tissue may be to enhance the defense structure of the leaf against the external environment.

The survival and growth of plants depend on the regulation of leaf stomata on carbon acquisition and water loss. The ability of species to maintain carbon balance largely depends on the difference in stomata response to drought stress [41]. Under the influence of impervious covers (such as cement, wood board), soil bulk density increases significantly and soil porosity decreases, making plants in an airtight and impervious environment for a long time. Therefore, plants may reduce leaf stomata density to reduce the loss of water and material through stomata to maintain normal water balance.

### Correlation of leaf functional traits and trade-off strategies under urban tree pit cover

Due to the pressure of the external environment, there will be a certain quantitative relationship between plant functional traits, forming a complex and orderly trade-off network of economics spectrum traits [42–45]. In this study, under the influence of different covers, urban greening plants showed obvious functional balance changes in leaf functional traits. There was a significant negative correlation between stomata density and stomata area, which was consistent with the research conclusions of many scholars [46–48]. This change was closely related to the self-protection of plants in the urban environment. In poorly water-permeable materials such as cement, soil often has poor air and water permeability. At this time, the density of stomata per unit leaf area of plants decreases due to the decrease in specific leaf area. Plants increase the stomata area of their leaves to maintain the balance of exchange between leaves and external substances.

In this study, there was a significant negative correlation between leaf thickness and stomata area. The purpose of increasing the leaf area of plants is to avoid the imbalance of leaf water drainage capacity caused by the increase in thickness. The specific leaf area was negatively correlated with leaf dry matter content and leaf tissue density. This fully shows that under the condition of soil compaction caused by tree pit cover, urban plants often use more synthetic substances to increase the construction of protective tissues to adapt to the coercive environment. When the water supply is insufficient, the specific leaf area becomes smaller, while the leaf tissue density and leaf dry matter content increase [49]. The leaf turnover growth rate of high tissue density slows down, and more nutrients are used for defense structure. Therefore, *Fraxinus chinensis* can form an adaptation strategy to the urban environment through the functional balance changes of specific leaf area, leaf tissue density, and leaf dry matter content.

**Fig. 5:**
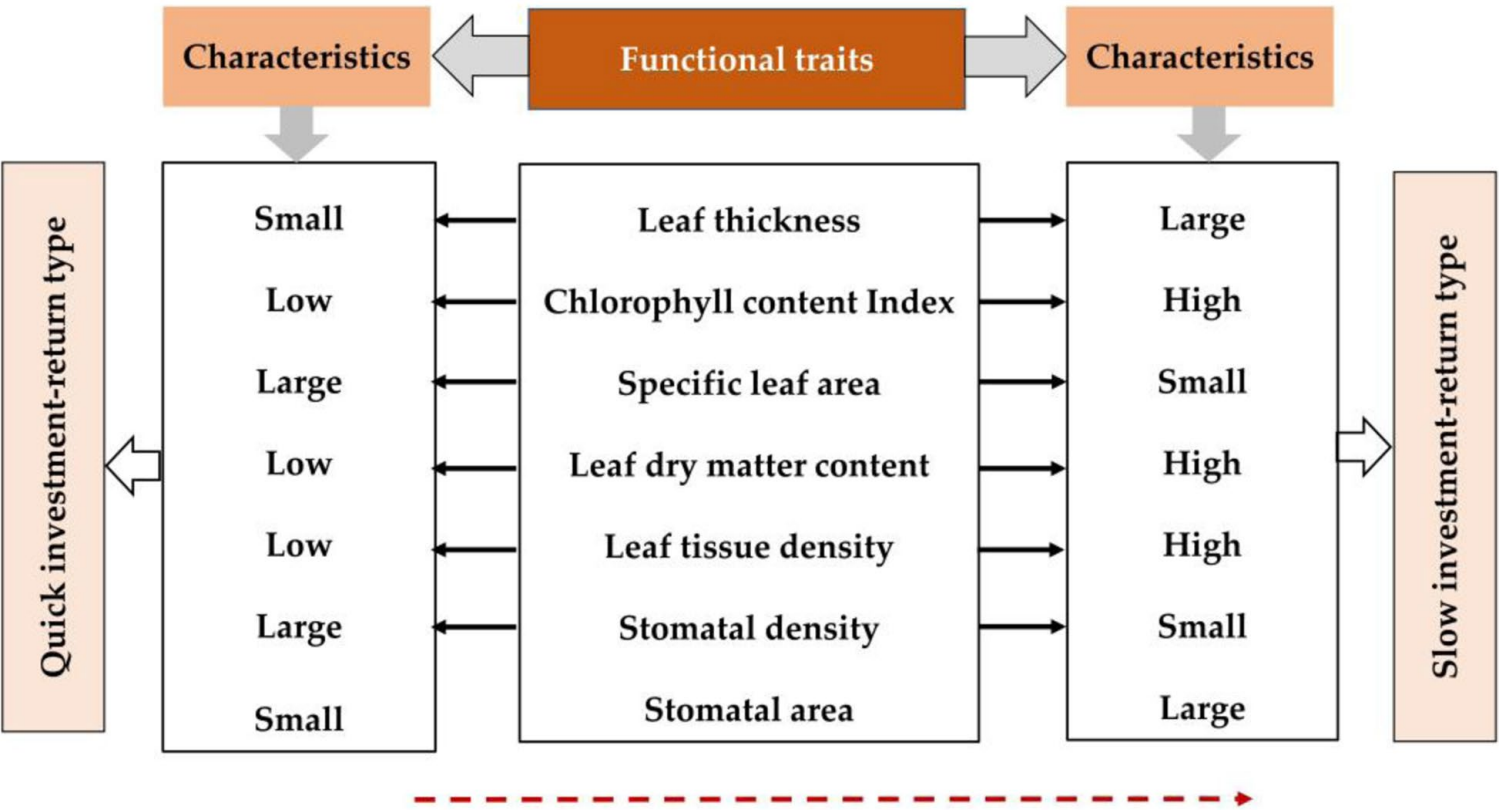
Leaf economics spectrum and their offset direction under the influence of tree pit covers

As a bridge connecting plants and the environment, plant functional traits play an important role in the study of the relationship between them [35, 44]. Leaf economics spectrum is a series of interrelated and coordinated changes of functional traits. At one end of this continuously changing functional trait portfolio spectrum is the quick investment-return type, and the other end is the slow investment-return type [44, 50, 51]. In this study, the different coverings in the urban tree pits promoted the functional adjustment of urban green plants at the leaf level to adapt to environmental changes and form the best combination of functions to achieve their own survival and reproduction. In short, this study takes urban greening plants as the research object, analyzes and verifies the existence of leaf economics spectrum in urban environment. As shown in Fig. 5, we found that plant leaves have low specific leaf area, stomata density, high leaf thickness, chlorophyll content index, leaf dry matter content, leaf tissue density and stomatal area, which were classified as “quick investment-return” type in the leaf economics spectrum.

## Conclusion

Overall, our findings indicate that there were significant differences in plant functional traits in the tree pits treated with different covering materials. Furthermore, we confirm that urban tree pit cover was an important factor affecting the change of urban tree functional traits. In this environment, plants adapt to the environmental changes by adjusting and changing their own leaf functional traits, and form different survival strategies such as growth, reproduction and defense. Results verify the existence of the leaf economics spectrum in the urban environment. Under the influence of urban tree pits coverage, plant leaves have low specific leaf area, stomata density, high leaf thickness, chlorophyll content index, leaf dry matter content, leaf tissue density and stomata area, which belong to “quick investment-return” type. The management of urban street trees, especially the construction of urban infrastructure, is vital to the growth of plants. This further supports the viewpoint that the influence of plant growth differences (especially the arrangement and selection of tree pits) should be considered in urban planning.

## Methods

### Study area

The study site is located in Zhengzhou City, Henan Province, China, between longitudes 112°42′ and 114°13′E and between latitudes 34°16′ and 34°58′N, and belongs to the continental monsoon climate in the northern temperate zone. The annual average temperature is 15.6 °C. August is the hottest month, with a monthly average temperature of 25.9 °C. January is the coldest, with a monthly average temperature of 2.15 °C. The annual average rainfall is 542.15 mm. The main greening tree species in the city are *Fraxinus chinensis* Roxb., *Sophora japonica* Linn., and *Ligustrum lucidum* Ait.

### Sampling method

**Fig. 6:**
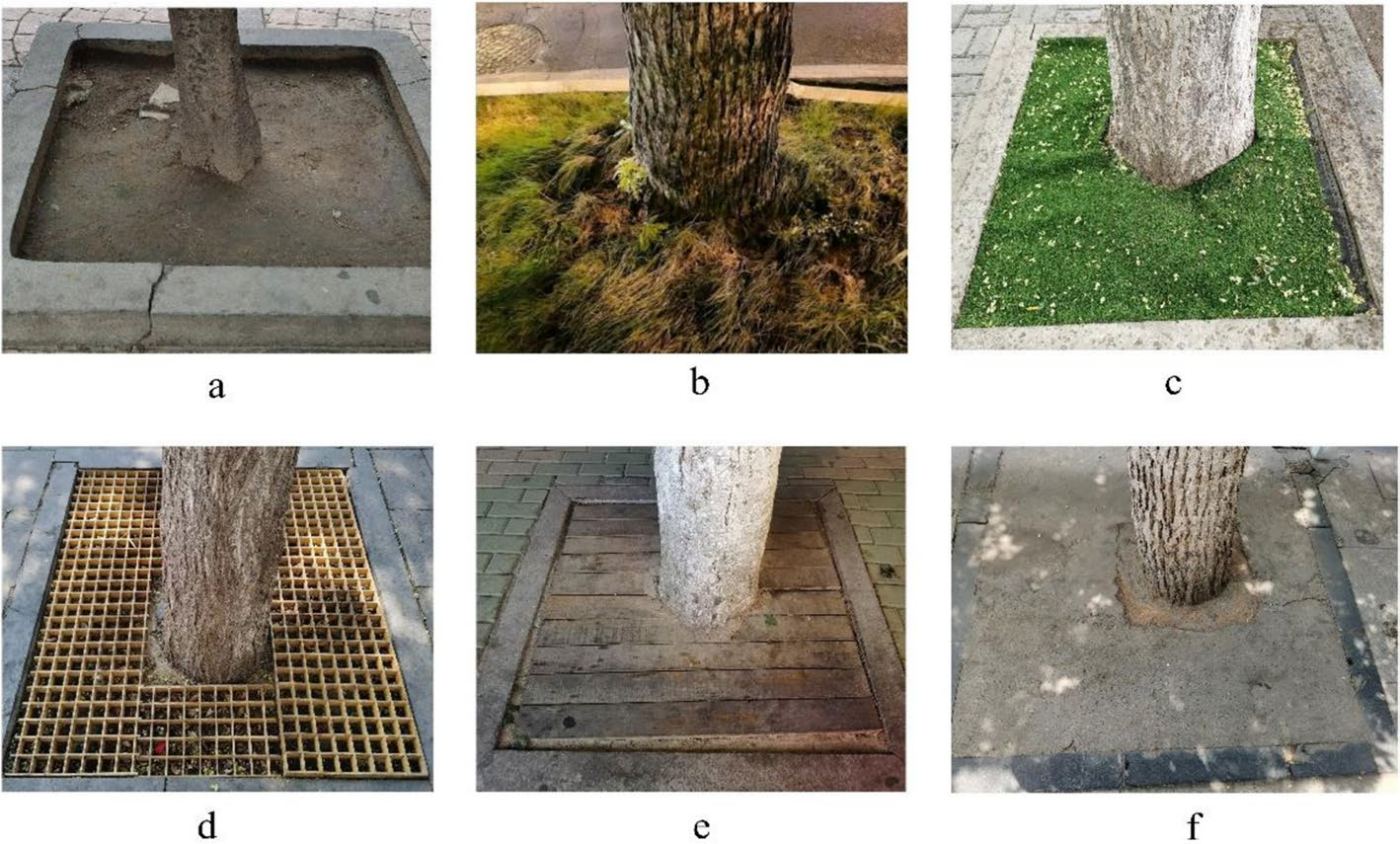
Six common types of covering materials for urban tree pits. **a** No cover (CK), **b** Turf (T1), **c** Breathable plastic blanket, **d** Tree grate, **e** Wood board, **f** Cement

This experiment was conducted in July 2020. As shown in Fig. 6, we selected six types of tree pit cover in the city: no cover (CK), turf (T1), permeable plastic blanket (T2), tree grate (T3), wood board (T4), cement (T5). The size of the tree pit is 1.5 m × 1.5 m. We choose the tree species commonly used in Zhengzhou City—*Fraxinus chinensis* Roxb. The time of trees being growing under the cover material is 15 years. The average height is 12.5 m, and the average diameter at breast height is 32.8 cm. Thirty trees were randomly selected for each treatment. Leaf samples were randomly collected from the middle canopy of the trees in the east, west, south, and north directions. Each tree randomly collected 30 mature, healthy leaves (8–10 leaves were collected in each direction). The leaf samples selected were all located on the same main road in Zhengzhou city, which ensures the relative consistency of environmental factors such as light, moisture, nutrients, temperature, etc. All the plant samples involved in this study were identified by Professor Chengyang Xu of Beijing Forestry University (refer to Flora of China). These voucher samples have been deposited in the Laboratory of Urban Forestry Research Center of Beijing Forestry University.

### Plant functional traits measurements

Before measuring the leaf trait index, we first use sanitary napkins to remove dust from the leaves surface. Fresh leaf quality (LFW) was weighed by JA1003N one-thousandth electronic balance (Shanghai Jinghai Instrument Co., Ltd., Shanghai), and all leaf samples were measured within 30 min after removal. The leaf area (LA) was measured by V370 portable leaf area meter (Seiko Epson Corporation, Shanghai, China), and the leaf area was automatically calculated based on the instrument’s own software (LEAFAREA). Leaf thickness (LT) was measured with 500–196-30 digital vernier calipers (Suzhou Quantum Instrument Co., Ltd., Suzhou, Jiangsu, China). The thickness of each leaf was measured at 3 equidistant points along the main vein, and the thickness values of the three positions were averaged as the leaf thickness of the leaf, repeated 3 times. Leaf volume (LV) was measured by drainage method. First, we put an appropriate amount of distilled water (V_1_) in a 500 mL graduated cylinder, and then immerse the leaf in water (V_2_). The difference between the two water volumes is the volume of the leaf (LV=V_2_-V_1_). Then, the leaves were soaked in distilled water and placed in a refrigerator at 5 °C in a dark environment for 12 h. Then the water on the surface of the leaves was absorbed with absorbent paper. The leaf saturated fresh weight (LSFW) was weighed. Finally, put the leaves into the GZX-GF101 blast drying oven (Shanghai Xin Yi Instruments and Meters Co., Ltd., Shanghai, China), and dry them at 60 °C to constant weight (48 h), and weigh the dry weight (LDM).

1 $$\mathrm{LTD}=\mathrm{LDM}/\mathrm{LV},\mathrm{g}/{\mathrm{cm}}^3$$

2 $$\mathrm{SLA}=\mathrm{LA}/\mathrm{LDM},{\mathrm{m}}^3/\mathrm{g}$$

3 $$\mathrm{LDMC}=\mathrm{LDM}/\mathrm{LSFW},\mathrm{g}/\mathrm{g}$$

The stomatal density was prepared by the blotting method. First, we evenly apply a layer of transparent imprinting liquid on the back of the blade, and then use tweezers to remove the imprinting film from the upper, middle, and bottom of the blade, and make a temporary glass slide. Three glass slides were made per leaf. Each glass slide was magnified by XSP-20 optical microscope (Jiangnan Yongxin Optics Co., Ltd., Jiangsu, Nanjing, China), and then randomly selected 5 fields of view (713.191 μm × 958.115 μm) for image acquisition. The stomatal density and stomatal area were calculated using the microscope’s own software (Scope Image 9.0).

### Soil physical properties

Choose a sunny day with no precipitation to collect soil samples (July 2020). Collect ring knife samples from 0 ~ 10 cm soil layer. When we collect soil under cement, we need to pry open three round holes with a diameter of 15 cm. Sixty soil samples were collected for each type of tree pit cover. Physical indicators such as soil bulk density, total porosity, capillary porosity and non-capillary porosity were measured by the ring knife method.

**Fig. 7:**
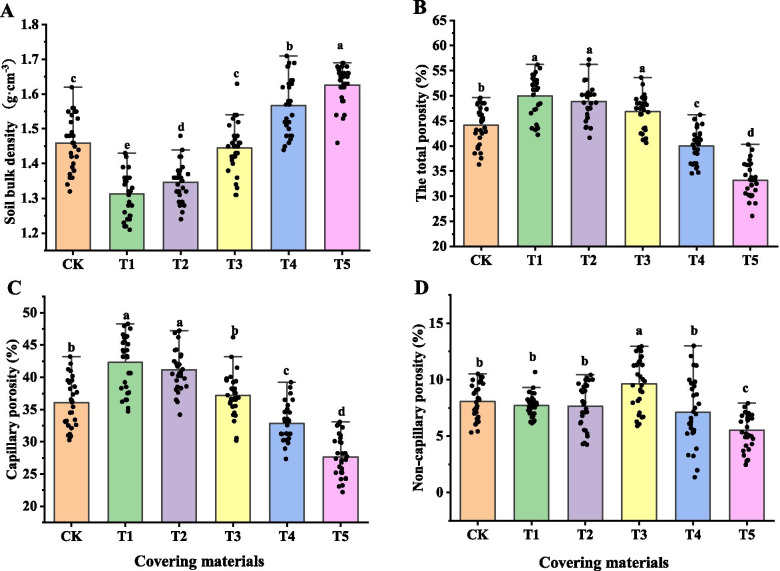
The effect of different covering materials on the physical properties of soil in urban tree pits. **A** Soil bulk density, **B** Total soil porosity, **C** Soil capillary porosity, **D** Soil non-capillary porosity. No cover (CK), turf (T1), permeable plastic blanket (T2), tree grate (T3), wood board (T4), cement (T5)

As shown in Fig. 7, the soil bulk density (0 ~ 10 cm) of urban tree pits under different covering materials was cement > wooden board > CK > tree grate > permeable plastic blanket > turf. The soil bulk density of the cement-covered tree hole was significantly higher than other treatments. The total porosity of urban tree pit soil under different covering materials was as follows: turf > permeable plastic blanket > tree grate > CK > wood board > cement. The soil capillary porosity of urban tree pits under different covering materials was as follows: turf > permeable plastic blanket > tree grate > CK > wood board > cement. The soil capillary porosity of the cement-covered tree pit was significantly lower than that of other treatments. The non-capillary porosity of urban tree pit soil under different covering materials was as follows: tree grate > CK > turf > permeable plastic blanket > wood board > cement.

### Data analysis

All data was sorted in Excel 2020, and Origin 2019b was used to analyze the data and make figures. One-way analysis of variance (ANOVA) and LSD multiple comparisons were used to test the significance of the differences in leaf functional traits among different urban tree pit cover types. Linear regression was used to analyze the relationship between leaf traits, and principal component analysis was used to comprehensively analyze the relationship between leaf traits and ecological strategy.

## Data Availability

The datasets generated during the current study are available from the corresponding author on reasonable request after consulting the project funder.

## Abbreviations

SLA: Specific leaf area
LDMC: Leaf dry matter content
CCI: Chlorophyll content index
LTD: Leaf tissue density
LT: Leaf thickness
LSFW: Leaf saturated fresh weight
LV: Leaf volume
LDW: Leaf dry weight
SD: Stomatal density
SS: Stomatal area
